# Towards the clinical translation of a silver sulfide nanoparticle contrast agent: large scale production with a highly parallelized microfluidic chip

**DOI:** 10.1007/s00259-024-06967-5

**Published:** 2024-11-12

**Authors:** Katherine J. Mossburg, Sarah J. Shepherd, Diego Barragan, Nathaniel H. O, Emily K. Berkow, Portia S. N. Maidment, Derick N. Rosario Berrios, Jessica C. Hsu, Michael J. Siedlik, Sagar Yadavali, Michael J. Mitchell, David Issadore, David P. Cormode

**Affiliations:** 1 Department of Bioengineering, University of Pennsylvania, Philadelphia, PA USA; 2 Department of Radiology, University of Pennsylvania, Philadelphia, PA USA; 3 Department of Biology, University of Pennsylvania, Philadelphia, PA USA; 4 Department of Pharmaceutical Sciences, St. Joseph’s University, Philadelphia, PA USA; 5 Department of Physics, St. Joseph’s University, Philadelphia, PA USA; 6 Department of Chemistry, University of Pennsylvania, Philadelphia, PA USA; 7 Biochemistry and Molecular Biophysics Graduate Group, University of Pennsylvania, Philadelphia, PA USA; 8 InfiniFluidics, Inc, Philadelphia, PA USA; 9 Cardiovascular Institute, Perelman School of Medicine, University of Pennsylvania, Philadelphia, PA USA; 10 Department of Electrical and Systems Engineering, University of Pennsylvania, Philadelphia, PA USA; 11 Department of Chemical and Biomolecular Engineering, University of Pennsylvania, Philadelphia, PA USA; 12 Departments of Radiology and Medical Physics, University of Wisconsin-Madison, Madison, WI USA

**Keywords:** Silver sulfide, Nanoparticles, Contrast agents, High throughput, Large scale synthesis

## Abstract

**Purpose:**

Ultrasmall silver sulfide nanoparticles (Ag_2_S-NP) have been identified as promising contrast agents for a number of modalities and in particular for dual-energy mammography. These Ag_2_S-NP have demonstrated marked advantages over clinically available agents with the ability to generate higher contrast with high biocompatibility. However, current synthesis methods for inorganic nanoparticles are low-throughput and highly time-intensive, limiting the possibility of large animal studies or eventual clinical use of this potential imaging agent.

**Methods:**

We herein report the use of a scalable silicon microfluidic system (SSMS) for the large-scale synthesis of Ag_2_S-NP. Ag_2_S-NP produced using this system were compared to bulk synthesis and a commercially available microfluidic device through characterization, contrast generation, in vivo imaging, and clearance profiles.

**Results:**

Using SSMS chips with 1 channel, 10 parallelized channels, and 256 parallelized channels, we determined that the Ag_2_S-NP produced were of similar quality as measured by core size, concentration, UV–visible spectrometry, and in vitro contrast generation. Moreover, by combining parallelized chips with increasing reagent concentration, we were able to increase output by an overall factor of 5,100. We also found that *in vivo* imaging contrast generation was consistent across synthesis methods and confirmed renal clearance of the ultrasmall nanoparticles. Finally, we found best-in-class clearance of the Ag_2_S-NP occurred within 24 h.

**Conclusions:**

These studies have identified a promising method for the large-scale production of Ag_2_S-NP, paving the way for eventual clinical translation.

**Supplementary Information:**

The online version contains supplementary material available at 10.1007/s00259-024-06967-5.

## Introduction

Inorganic nanoparticles are being explored for many biomedical applications and have been identified as valuable options for improving disease detection in clinical imaging [1–4]. Their tunable properties, such as photon attenuation, size, shape, and surface charge, enable adaptation for a wide variety of imaging modalities and disease settings [5–7]. Therefore, much attention has been focused on designing inorganic nanoparticles for improved cancer detection and monitoring.

Recent work has identified sub-5 nm glutathione-coated silver-sulfide nanoparticles (Ag_2_S-NP) as a promising imaging contrast agent that is especially well-suited for breast cancer screening due to their strong x-ray attenuation in the energy range used for mammography [8–13]. Ag_2_S-NP have demonstrated improved contrast over traditional iodinated agents and have a longer circulation window [8]. Furthermore, they can be synthesized at room temperature, under ambient conditions, and in water, while other inorganic nanoparticles require high temperatures and toxic reagents for synthesis [2, 14–16]. Additionally, silver-based nanomaterials are much less expensive than their gold counterparts, making them more practical economically [17]. Previous studies have shown that Ag_2_S-NP also are highly biocompatible, both in vitro and in vivo [8–10, 18]. In particular, Ag_2_S-NP had no significant impact on the viability of healthy liver and kidney cells in vitro, which are expected to have the most contact with the contrast agent [8]. Due to their ultrasmall size, these Ag_2_S-NP are renally excretable, which is an improvement over many alternative inorganic nanoparticle formulations and makes them appropriate for eventual clinical translation [7, 19–21]. However, to facilitate large animal studies and clinical translation of Ag_2_S-NP, a rapid and reliable synthesis method must be established.

Current synthesis methods available for Ag_2_S-NP either produce nanoparticles of variable physical characteristics or in quantities that are too small for clinical relevance [22–24]. Bulk synthesis methods permit larger volumes and scale but suffer from temperature and concentration gradients that result in nanoparticles with heterogenous size distributions. Although previous work has shown that Ag_2_S-NP size can be controlled in bulk synthesis using high temperatures, an inert atmosphere and organic solvents, each of which are problematic for large scale synthesis of pharmaceuticals [8]. Additionally, because bulk methods rely on batch processes, there is variability amongst various runs of the synthesis. Microfluidic mixing techniques, such as staggered herringbone mixing, are highly valuable tools for decreasing dispersity, controlling size without high temperatures or organic solvents, and ensuring reproducibility compared to bulk mixing; however, they have been limited by low throughputs [25–27]. The previously reported microfluidic synthesis method for Ag_2_S-NP produces approximately 20 mg of product (by silver mass) per hour, which then must be concentrated and purified [10]. At this rate, it would take over two weeks to produce one dose for the average American woman for the synthesis alone, based on a moderate dose of 100 mg Ag/kg [28].

**Fig. 1:**
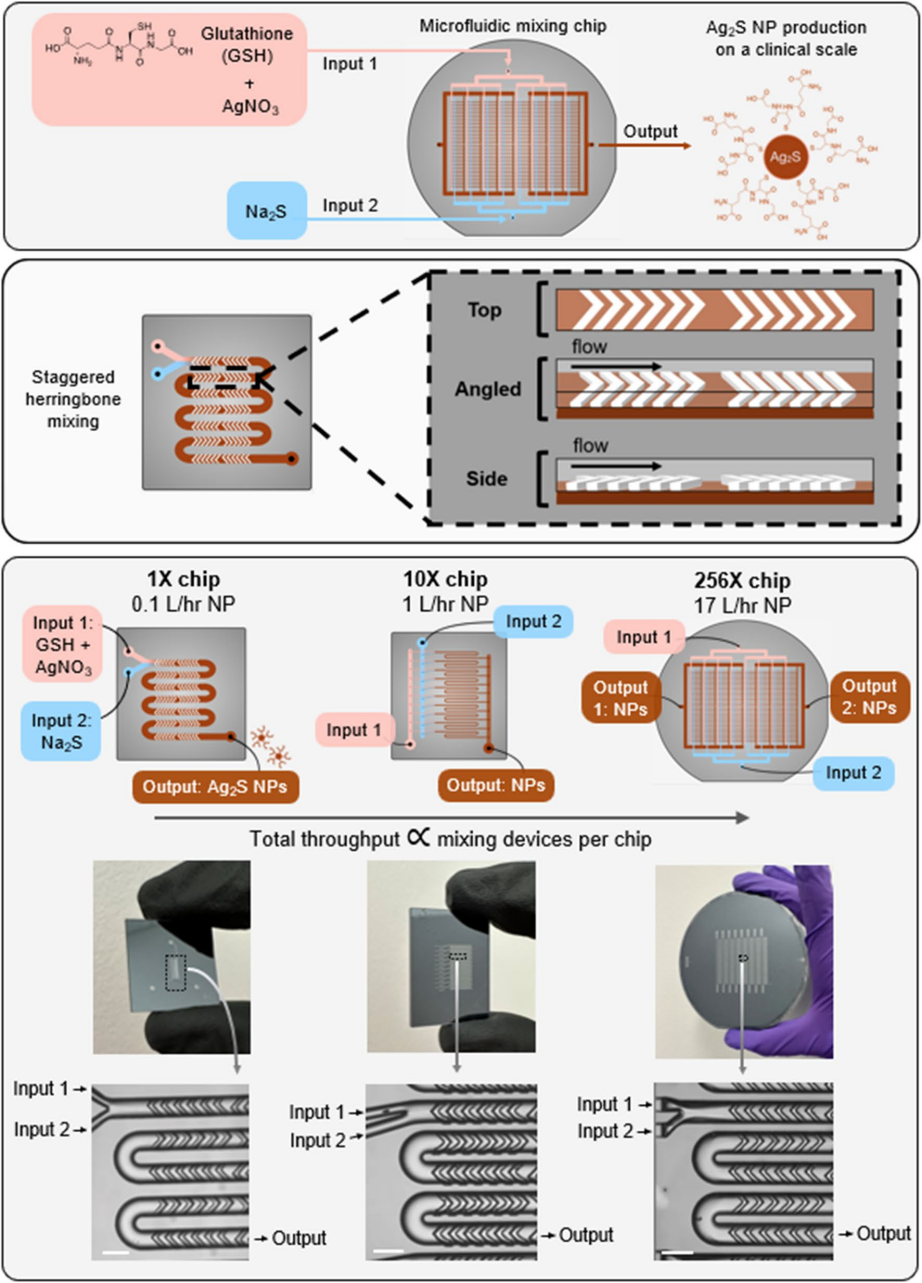
Schematic, photographs, and micrographs showing microfluidic chips used for synthesis of Ag_2_S-NP

To overcome these limitations, this study sought to evaluate Ag_2_S-NP production using a scalable silicon microfluidic system (SSMS) to confirm that nanoparticles are of the same quality as those produced on a small scale. We use aqueous solvents, room temperature and ambient conditions, which is attractive for large scale production. SSMS is a novel microfluidic platform which allows for solutions to flow through multiple parallelized staggered herringbone mixing channels simultaneously. In this study, we focused on SSMS with 1 channel, 10 parallelized channels, and 256 parallelized channels (Fig. 1). With only a single set of inputs and outputs, for delivery of reagents and collection of nanoparticles, respectively, the SSMS allows for the logistical ease of operating only one device with the production power of many. The unique design of this platform includes flow resistors which ensure equal flow rates in each parallel channel, resulting in identical product synthesis across the chip [29]. The materials used in the fabrication of SSMS were selected to meet industry standards for pharmaceutical production, paving the way for eventual translation [30]. Previous work with a system similar to SSMS has indicated that it is possible to produce high-quality nanomaterials at a drastically increased rate, but these studies were done with organic lipid nanoparticles [29, 31–34]. A microfluidic device designed for large-scale production of Ag_2_S-NP will generate consistent batches at high production rates, enabling the clinical studies necessary for translation to patient care. Therefore, the studies presented here are on the forefront of advancing microfluidic devices for clinical scale production of inorganic nanoparticles.

Herein, we report the rapid synthesis of monodispersed, sub-5 nm Ag_2_S-NP using the SSMS microfluidic chip with up to 256 parallel channels while monitoring the chip and the product characteristics. We evaluated the nanoparticle quality through the size and absorption spectra while increasing the production rate from 0.1 L/hr to 17 L/hr. Moreover, we studied the effect of increasing reagent concentrations on Ag_2_S-NP characteristics and yield. The nanoparticles were also studied in phantom and mouse models for their contrast generation and compared to previous studies and clinically available agents. Additionally, the biodistribution of remaining silver in mouse models was measured to determine 24-h clearance rates of Ag_2_S-NP from each synthesis method.

## Materials

Silver nitrate (AgNO_3_, 99%), sodium sulfide (Na_2_S, 98%), and L-glutathione (GSH, 98%) were purchased from Sigma-Aldrich (St. Louis, MO). Sodium hydroxide (NaOH) was purchased from Fisher Scientific (Pittsburgh, PA). A commercially available staggered herringbone single channel microfluidic chip was purchased from Microfluidic ChipShop (Jena, Germany). Iopamidol was obtained from Bracco Diagnostics (Monroe, NJ). Nude mice were acquired from The Jackson Laboratory (Bar Harbor, ME).

## Methods

### Fabrication of SSMS chips

Microfluidic chips were fabricated in silicon and glass substrates as previously described [35] using a process of four-layer lithography combined with ion etching to fabricate 3D structures. This 3D architecture distributes the input reagents uniformly to each individual mixing unit where the nanoparticles are formulated, then the nanoparticles are collected in a pooled output. Briefly, a single 100 mm double-sided polished silicon wafer (University Wafer, South Boston, MA) is lithographically patterned with S1805 photoresist to define the features, then etched to the intended etch depth using deep reactive ion etching. This process of lithography/etching is repeated for all four layers. The etched silicon wafer is anodically bonded to glass wafers on each side to encapsulate the microfluidic channels. Inputs and outputs are connected to the microchannels by machined holes patterned in one of the glass wafers. The bonded chip is fabricated in the Quattrone Nanofabrication Facility at the University of Pennsylvania. Chips were fabricated with one mixing channel (1X SSMS), 10 parallelized mixing channels (10X SSMS), and 256 parallelized mixing channels (256X SSMS).

To operate the silicon and glass chips, a custom pressure-driven flow system was engineered as previously described [35]. Briefly, the two inputs are housed in pressurized vessels which are connected to a nitrogen tank and controlled by pressure controllers to regulate the input flow rates. The silicon and glass chip is encased in an aluminum housing system to connect to the input/output tubings. Chip performance is monitored by a DM4 B upright microscope (Leica Microsystems GmbH, Germany) to ensure a 3:1 flow rate ratio between the GSH + AgNO_3_ and Na_2_S inputs, respectively. For this study, the maximum total flow rate used was 2 mL/min/channel for 1X and 10X SSMS and 1.1 mL/min/channel for 256X SSMS, but previous studies have included total flow rates of up to 2.4 mL/min/channel [29].

### Synthesis of Ag_2_S-NP

Ag_2_S-NP were synthesized according to a protocol adapted from previous reports [8, 10]. The basic protocol is as follows: two solutions were prepared and co-injected into a staggered herringbone microfluidic mixing chip, either commercially purchased or fabricated as described previously. One solution was prepared with 767 mg GSH and 42.5 mg AgNO_3_ in 75 mL of deionized water, then the pH of this solution was adjusted to 7.4 using NaOH. For the second solution, 10 mg of Na_2_S was dissolved in 25 mL of water. The two solutions were co-injected into a mixing chip at a 3:1 rate, respectively, with an overall flow rate of 2 mL/min/channel. Due to limitations of the experimental setup, the 256X SSMS chip was operated at a total flow rate of 17 L/hr (= 1.1 mL/min/channel). The SSMS system was set up to use a pressure-driven system to drive the flow rate, but was only safe for operation up to 100 PSI. In the event that this system is used regularly with the 256X chip, the flow system could be adjusted to accommodate the 200 PSI that would theoretically be necessary to operate at a total flow rate of 31 L/hr. Pressure controllers, installed in the input vessels, were used to modify pressure in these containers, which drove the flow rate of the input solutions. The output was collected and then concentrated and washed with DI water using 3 kDa molecular weight cut-off (MWCO) filtration tubes (Sartorius Stedim Biotech, Germany). The tubes were centrifuged at 4000 rpm in 20-min cycles until the final output was concentrated 100-fold. The concentrated nanoparticles were then filtered through a 0.020 µm filter membrane (Whatman Anotop, Boston, MA) and stored at 4 °C until use in experiments.

The Ag_2_S-NP from a commercially available chip (CS-Ag_2_S-NP) were prepared according to the protocol described here using the Fluidic 187 Micro Mixer from the Microfluidic ChipShop (Jena, Germany). Ag_2_S-NP were each prepared using the 1X, 10X, and 256X SSMS chips (1X-Ag_2_S-NP, 10X-Ag_2_S-NP, and 256X-Ag_2_S-NP respectively). Bulk-Ag_2_S-NP were synthesized by combining the two previously described solutions in a beaker and allowing the mixture to stir for 20 min. After this, the solution was concentrated, washed, and filtered as described above.

Increased reagent concentrations were also used for the synthesis of Ag_2_S-NP. For these studies, the same volume of water was used to prepare each solution, however the reagent concentrations used were 100%, 500%, 1000%, 2000%, and 3000% of the originally stated concentrations. The synthesis was then run and followed by concentration and purification as previously described.

### UV/visible absorption

All Ag_2_S-NP were characterized using a Thermo Scientific Genesys UV/Visible Spectrophotometer to record the absorbance of each sample. Samples were prepared by diluting Ag_2_S-NP stock solutions in deionized water to 10 µg/mL and adding 1 mL to a cuvette for analysis. Scans were performed between 350 and 1000 nm wavelengths.

### Transmission electron microscopy

Each sample was analyzed with transmission electron microscopy (TEM) using a T12 cryo-electron microscope (FEI Tecnai) operated at 100 kV. Samples were prepared by dropping 10 µL of diluted samples of Ag_2_S-NP onto formvar-coated, carbon-film stabilized copper grids (Electron Microscopy Sciences, Hatfield, PA) and allowing them to air dry. ImageJ (National Institutes of Health, Bethesda, MD) was used to measure the core diameter of 100 nanoparticles per sample. A one-way ANOVA test with Turkey’s multiple comparisons was used to identify significant differences.

### Inductively coupled plasma optical emission spectroscopy

The concentration of silver in each sample was measured using Spectro-Genesis inductively coupled plasma – optical emission spectroscopy (ICP-OES). Samples were prepared by dissolving 10 µL of Ag_2_S-NP in 1 mL of nitric acid and then diluting with deionized water to a final volume of 10 mL. Each Ag_2_S-NP solution had samples prepared in triplicate.

### Phantom imaging

Samples for phantom imaging of each type of contrast agent were prepared in triplicate with concentrations of 0, 0.5, 1, 2, 4, 6, 8, and 10 mg/mL. Each sample was loaded into a 0.2 mL tubes, placed in a rack, and covered in parafilm. Each rack of samples was scanned using a MILabs micro-CT scanner with tube voltage of 55 kV and isotropic 100 micron voxels with the following parameters: matrix size = 512 × 512, field of view = 37 × 37 cm, and reconstructed slice thickness = 0.5 mm. Attenuation rates for each contrast agent were determined using Osirix MD software and a one-way ANOVA with Tukey’s multiple comparisons test was used to determine statistical significance.

### Animal model

In vivo imaging was performed using 8 week-old female nude mice (Jackson Laboratories, Bar Harbor, ME) with 5 mice per group in accordance with protocols approved by the Institutional Animal Care and Use Committee of the University of Pennsylvania. As this study is based on breast cancer, and 99% of cases occur in women [36], female mice were used in accordance with NIH guidelines. Mice were scanned pre-injection, then administered Ag_2_S-NP at a dose of 250 mg Ag/kg body weight of the mouse via the tail vein. This dose is consistent with previous studies of this nanoparticle, as well as other similar heavy metal-based nanoparticles [8–10, 37–43]. Tail vein injection was selected to rapidly administer the agent directly into the bloodstream and most closely mimic clinical practice of intravenous administration. Mice were anesthetized for scans using isoflurane.

### In vivo imaging

All mice were imaged using a MILabs micro-CT scanner (tube voltage = 55 kV) with scans performed at 5, 30, 60, 120, and 1440 min post-injection. Parameters for the scans were as follows: matrix size = 512 × 512, field of view = 37 × 37 cm, reconstructed slice thickness = 0.5 mm. Scans were analyzed using Osirix MD and the change in attenuation from pre-scan to each measured time point is shown in Hounsfield units as mean ± SEM.

### Biodistribution

All mice were sacrificed 24 h post-injection using carbon dioxide inhalation and were dissected to collect relevant organs. For this study, the heart, lungs, liver, kidneys, spleen, blood, bladder, fecal matter, and tail were isolated, and the remainder of the carcass was collected. Each component was weighed and recorded before being digested in 4 mL of nitric acid for 24 h at 90 °C. 6 mL of deionized water was added to each sample and then they were analyzed using ICP-OES to detect silver content. Data is presented as mean ± SEM.

### Statistical analysis

All experiments described herein were performed in triplicate, unless otherwise specified. Statistical analysis was performed using GraphPad Prism 9 software with selected statistical tests described in the methods for each experiment.

## Results

### Synthesis and characterization

**Fig. 2:**
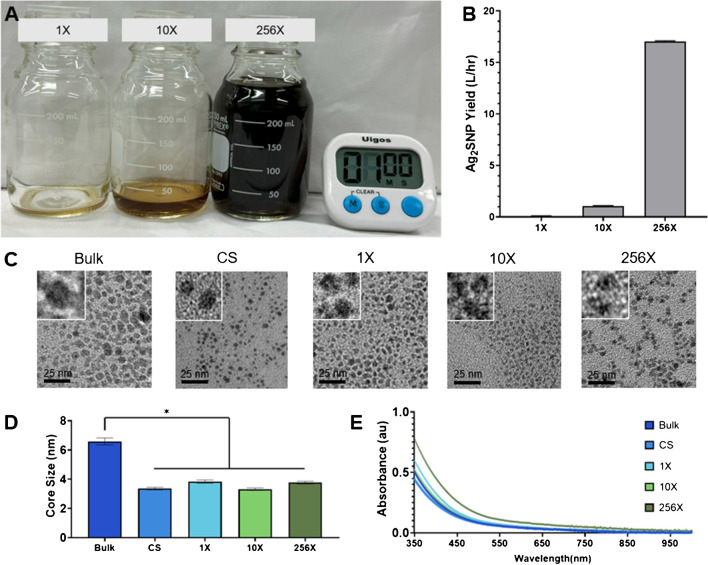
**A**) Representative images of Ag_2_S-NP produced using each SSMS chip after running for one minute and **B**) rate of production using each device (mean ± SEM). Characterization of the Ag_2_S-NP synthesized including **C**) representative TEM micrographs with insets showing 4 times the magnification, **D**) core size measurements (mean ± SEM), and **E**) UV–visible spectra

Ag_2_S-NP were synthesized using the bulk method, a commercially available microfluidic mixing chip, the 1X, 10X, and 256X SSMS chips. All SSMS chips were monitored during synthesis. While the 1X SSMS chip can produce only 0.1 L/hr of Ag_2_S-NP, the 10X SSMS chip produces Ag_2_S-NP at a rate of 1 L/hr and the 256X SSMS chip produces Ag_2_S-NP at 17 L/hr (Fig. 2a-b). In practice, this results in the ability to synthesize 100 mL of Ag_2_S-NP in 1 h (1X SSMS), 6 min (10X SSMS), or 23 s (256X SSMS), demonstrating a drastically increased rate of synthesis with increased parallelization. Ag_2_S-NP were found to be unchanging in core size and yield when isolated at various time points over five hours of microfluidic chip usage, confirming that output was of consistent quality and the chips continue to perform as expected (Fig. S1).

Using TEM, the diameters for the CS-, 1X-, 10X-, and 256X-Ag_2_S-NP were 3.3 ± 0.8 nm, 4.0 ± 0.6 nm, 3.4 ± 0.6 nm, and 4.4 ± 0.8 nm, respectively. The Bulk-Ag_2_S-NP had a core diameter of 6.6 ± 1.7 nm which is significantly larger than all the Ag_2_S-NP produced with microfluidic chips (Fig. 2c, d). The core sizes of each microfluidic chip-produced formulation indicate suitability for renal clearance *in vivo*, while the bulk synthesis did not produce Ag_2_S-NP that were sufficiently small enough for renal clearance, which illustrates the value of the use of the microfluidic chips. UV–visible absorbance spectra were also very similar for Ag_2_S-NP made via the five synthetic methods, which confirms the formation of Ag_2_S-NP as expected (Fig. 2e) [10]. The absorbance spectra here show the broad absorption band that is characteristic of these semiconducting quantum dots [8, 9, 44]. Further investigation using UV–visible spectroscopy revealed no change in the absorbance spectra after concentration and washing, indicating that no aggregation results from this process (Fig. S2). Zeta potential measurements indicated that all of the Ag_2_S-NP samples produced had highly negative surface charges, which are indicative of nanoparticle stability in solution (Fig. S2) [45]. Zeta potentials of greater than about 30 mV absolute value are expected to be stable, so these values confirm this feature in all of the Ag_2_S-NP studied here [46, 47]. X-ray diffraction patterns from each method are consistent with the monoclinic crystalline structure characteristic of Ag_2_S-NP (Fig. S3). X-ray photoelectron spectroscopy of all of the Ag_2_S-NP were also collected and displayed peaks for silver and sulfur (Fig. S5). The FT-IR spectra were similar across each synthetic method, which further confirms the comparability of these nanoparticles (Fig. S6). HR-TEM micrographs revealed similar lattice fringe distances to previous work, which are consistent with the monoclinic Ag_2_S phase (Fig. S7) [8].

**Fig. 3:**
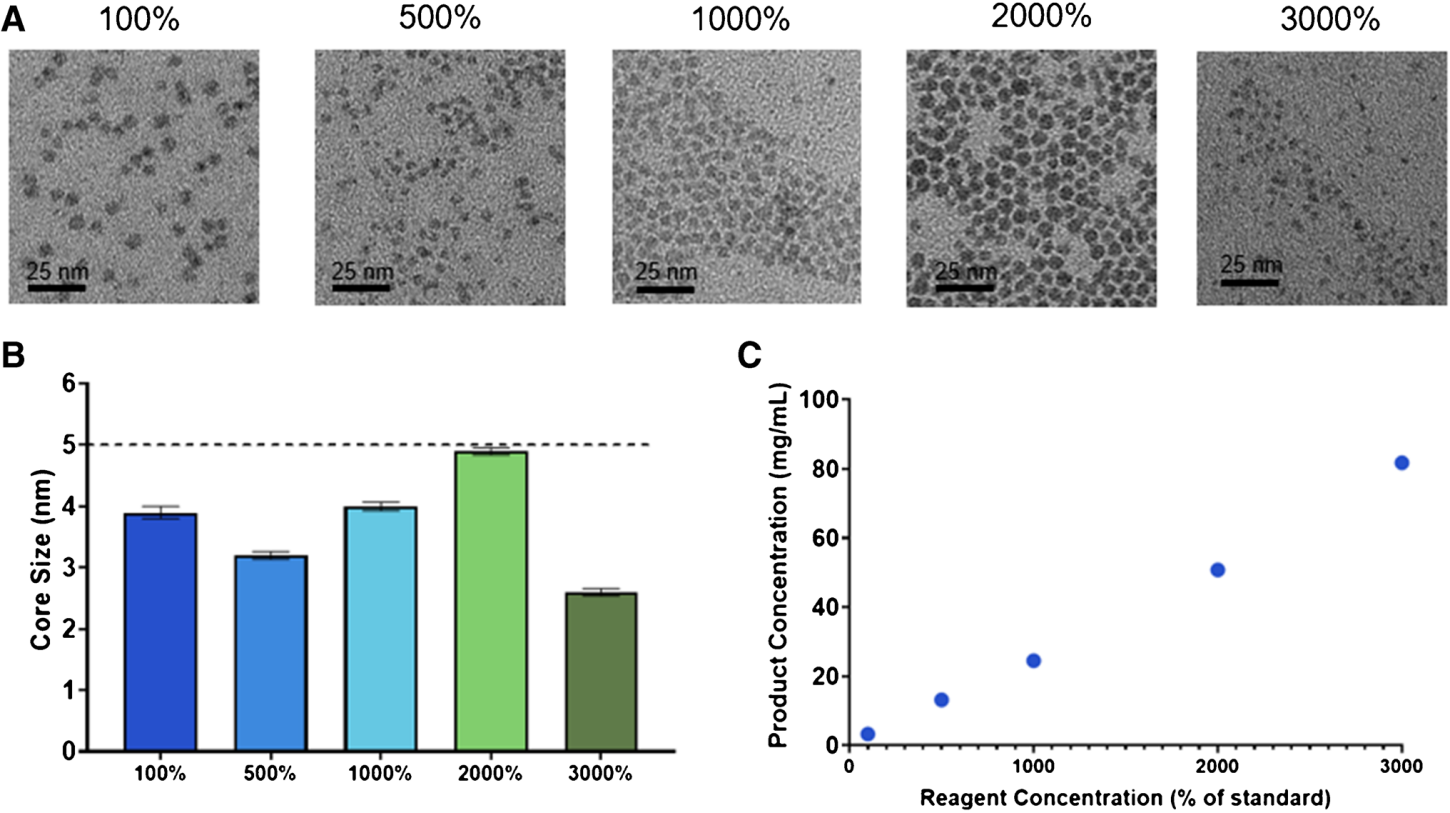
Characterization of the Ag_2_S-NP synthesized with increased concentrations including **A**) representative TEM micrographs, **B**) core size measurements (mean ± SEM), and **C**) product concentration as measured by ICP

To further scale up the process, we studied the effect of increasing the concentration of the reagents used. This would not only further decrease the time needed for synthesis and filtration, providing a higher production rate, but would also increase the reaction product concentration, reducing the total time needed for post-processing. To this end, Ag_2_S-NP were synthesized with standard conditions as well as reagents at 500%, 1000%, 2000%, and 3000% concentrations. The resulting nanoparticles were all found to be less than 5 nm, meeting the requirement for renal clearance, with no substantial differences in the diameters of the product between the conditions (Fig. 3a-b). Additionally, the yield of Ag_2_S-NP was found to scale linearly with increased concentrations, indicating that production does not diminish relative to the increased concentrations (Fig. 3c). The 3000% concentration studied here is near to the solubility limit for the reagents in water, so it is anticipated to be the highest reagent concentration available, and it did not result in any microfluidic chip clogging. These results suggest that increased concentrations of reagents may also be a valuable avenue for scaling up the synthesis methods of Ag_2_S-NP.

### In vitro contrast generation

**Fig. 4:**
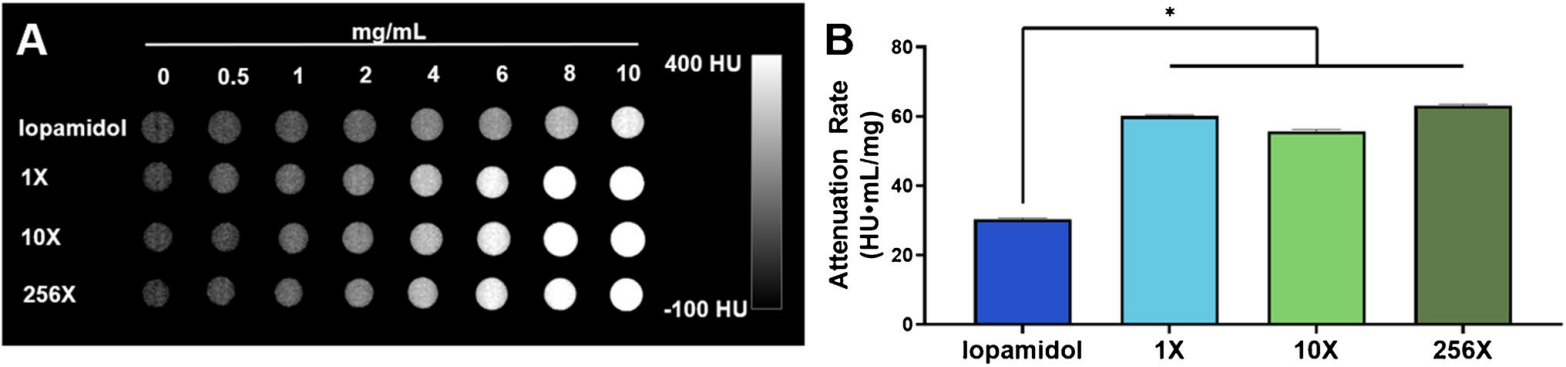
In vitro CT imaging. **A**) Representative µCT scans with iopamidol, 1X-Ag_2_S-NP, 10X-Ag_2_S-NP, 256X-Ag_2_S-NP at concentrations ranging from 0 – 10 mg/mL and **B**) quantification of the CT attenuation rate for the different solutions (mean ± SD)

Computed tomography (CT) scans of the phantoms prepared using each of the Ag_2_S-NP samples synthesized with an SSMS chip revealed no significant difference in attenuation rates as measured by an MI Labs microCT scanner. All SSMS chips produced Ag_2_S-NP with attenuation rates of about 60 HU·mg/mL with no significant difference among them. Importantly, the attenuation rates of the SSMS-synthesized Ag_2_S-NP were significantly higher than that of iopamidol, which only had an attenuation rate of 30 HU·mg/mL in this scanner (Fig. 4).

### In vivo imaging

**Fig. 5:**
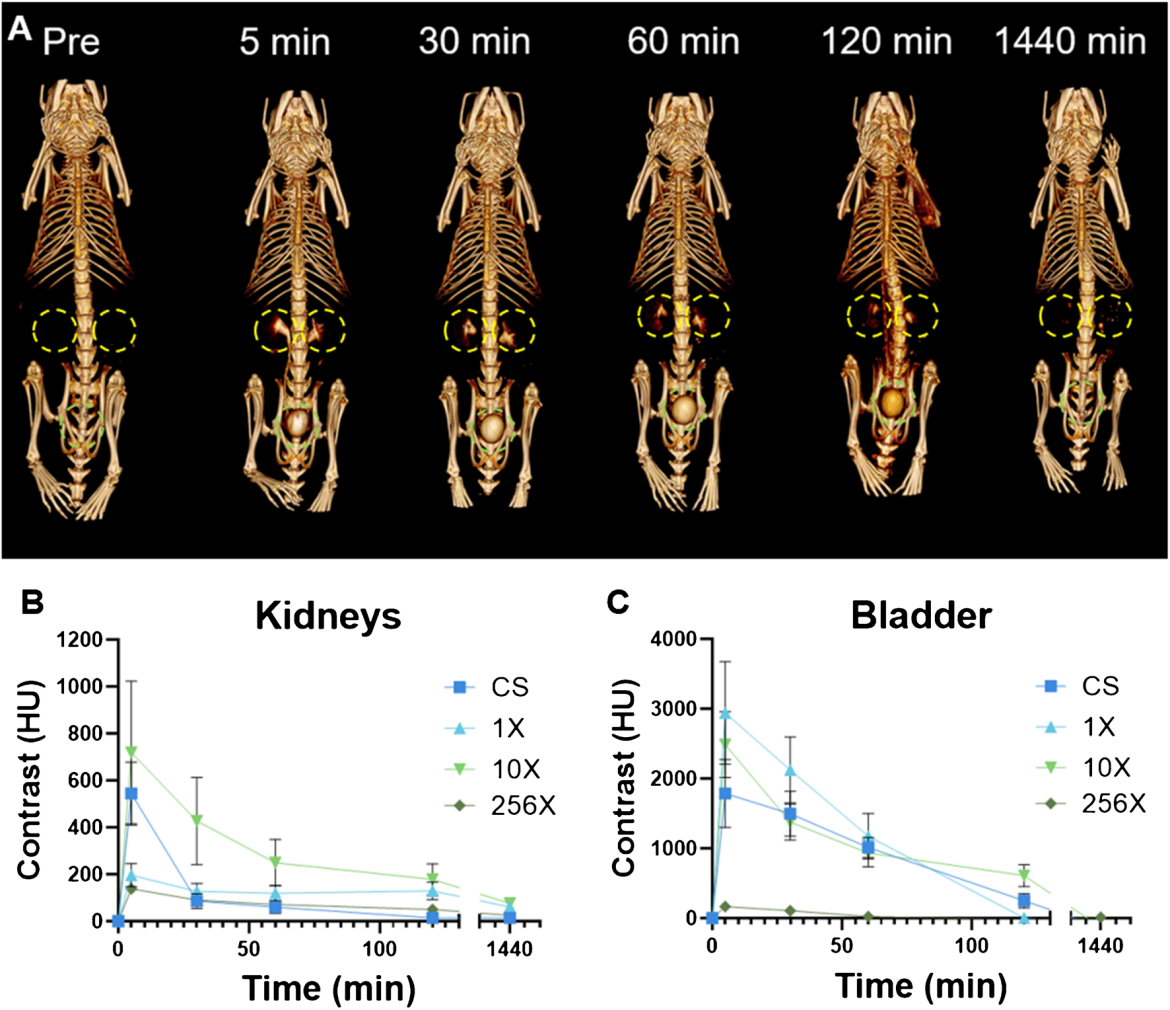
**A**) Representative 3D µCT images showing 10X-Ag_2_S-NP being renally cleared. Images include pre-injection and 5 min, 30 min, 60 min, 120 min, and 1440 min post-injection. Kidneys are indicated by yellow markers and bladders are indicated with green. Quantification of CT attenuation in the **B**) kidneys and **C**) bladder at each time point. *n* = 5 per group. Data is presented as mean ± SEM

Since the key criterion for clinical translation of an imaging agent containing a heavy element such as silver is excretion, we studied their renal clearance in mice via CT imaging. After intravenous injection with Ag_2_S-NP, CT scans revealed that Ag_2_S-NP prepared with each type of microfluidic chip were similarly excreted primarily through the renal clearance route. Visibly identifiable contrast is present in the kidneys and bladder beginning only 5 min after injection, continues to be noticeable through the 2-h time point, but is mostly eliminated by the 24-h scan (Figs. 5a, S8). Quantitative analysis of the scans confirms that increased contrast is present in the kidneys and bladder at 2 h post injection, but mostly eliminated by 24 h (Fig. 5b-c). Virtually no contrast was observed in the heart or liver (Fig. S9). These results were consistent across the CS-Ag_2_S-NP, 1X-Ag_2_S-NP, 10XAg_2_S-NP, and 256X-Ag_2_S-NP, and the variation in results can be attributed to varying times of excretion for each mouse.

### Biodistribution

**Fig. 6:**
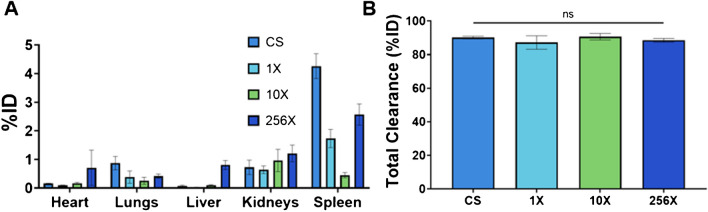
**A**) Biodistribution of Ag_2_S-NP in mice 24 h post-injection (mean ± SEM). n = 5 per group. **B**) Total clearance of Ag_2_S-NP in mice within 24 h (mean ± SEM)

24 h after the injection of Ag_2_S-NP, mice were sacrificed and their organs were collected for quantitative analysis silver content with ICP-OES. Analysis revealed very low retention of these nanoparticles, indicating that they were extensively renally cleared. The highest percentage of injected dose (%ID) was found in the spleen and kidneys, indicating primary renal clearance, with very small amounts found in RES organs. Carcass, including the tail, retention can be primarily attributed to the injection method (Fig. S10). Although there are some variations in uptake per organ between the synthesis methods, the uptake is very low relative to the total injected dose. Importantly, all synthetic methods resulted in around 90% clearance of the injected dose within 24 h with no significant difference between them (Fig. 6).

## Discussion

The results presented herein demonstrate a drastic capacity for increased synthesis without sacrificing particle quality, tunability, or imaging contrast generation. Compared to the more than 387 h needed to produce one human dose of the Ag_2_S-NP with a single channel microfluidic chip, using the 256X SSMS, one dose could be produced in 2.3 h. Coupled with 3000% concentration of reagents used, which was also demonstrated to be an effective method for increasing production, the synthesis could be completed in less than 5 min, representing scale-up of 5100-fold that could result in the production of > 8 doses per hour by a single chip. This impressive increase creates a feasible path for clinical level synthesis of Ag_2_S-NP, since additional avenues for further production increases remain, such as increasing the parallelization of the chip (e.g. to 1000 channels), running multiple chips at once, and increasing flow rates.

The SSMS were designed to comply with current clinical manufacturing standards and are made entirely out of silicon and glass to facilitate compatibility with many solvents and have high temperature tolerance, allowing for sterilization [31, 32]. Their silicon and glass composition also allow them to tolerate pressures up to 1000 PSI and can accommodate thousands of channels as needed. The geometry of the chip was designed to be resilient against clogging since the failure of any one channel does not affect production from the rest. Additionally, these materials allow for the resetting and reuse of these chips repeatedly [31]. Because of their compatibility with manufacturing standards, the SSMS system is a powerful tool for scaling up nanoparticle production.

Our synthesis methods using SSMS chips consistently resulted in sub-5 nm nanoparticles. Moreover, size can be further tuned by adjusting the relative flow rates of the reagents [10]. The contrast generation rates of the resulting Ag_2_S-NP are substantially improved over current clinical agents. We demonstrated potent in vivo contrast generation compared to commercial standards and a high safety profile. Previous work with Ag_2_S-NP confirms their safety profile through in vitro assays focused on hepatic and renal cells [8]. Combined, this points to the clinical potential of Ag_2_S-NP and the possibility of its use in place of iodinated agents in some applications [48]. For example, implementation of Ag_2_S-NP in the clinical paradigm of breast cancer imaging can provide better screening and earlier detection in high-risk populations, such as those with dense breast tissue [49, 50]. In this application, imaging would rely on the agent circulating and providing temporary contrast where extensive vasculature exists, as is a hallmark of a tumor [51]. Rapid, high renal clearance of Ag_2_S-NP, as indicated by high contrast in the kidneys and bladder shortly after injection, enhances the safety profile of this agent. Furthermore, this low-cost agent will be easy to implement due to its compatibility with imaging modalities that are readily available in the clinic such as mammography.

This study has also confirmed that the sub-5 nm Ag_2_S-NP produced using microfluidic synthesis methods have best-in-class renal clearance [8]. With a 24-h clearance of about 90% of injected dose for all microfluidic systems tested, Ag_2_S-NP have some of the highest clearance rates compared to other similarly sized metallic nanoparticles [52]. The clearance results displayed here are consistent with previous work with Ag_2_S-NP evaluating their clearance, which found an 85% to 93% clearance rate in 24 h [8, 10]. It is worth noting that this study was done in female nude mice in order to be consistent with NIH guidelines for studies involving breast cancer. While this study focused on demonstrating the renal clearance of Ag_2_S-NP, other studies have shown that similar NP accumulate in tumors, therefore we expect that a similar result would be seen with the Ag_2_S-NP [53]. Further, previous work has shown that Ag_2_S-NP can be used to image vasculature [8]. An interesting point to study in future work would be whether there is a difference in the biodistribution and clearance behavior of Ag_2_S-NP between wild type mice and immunocompromised nude mice. In addition, validation of the imaging properties of these agents in models of breast cancer would be valuable, although previous work with similar nanoparticles, including silver sulfide and silver telluride, has already shown high x-ray contrast in tumor models [9, 40].

Across each metric evaluated here, i.e. nanoparticle size, absorption spectra, imaging contrast in vitro and in vivo, and biodistribution, each SSMS synthesis platform produced nanoparticles of consistent quality and performance. This confirms the potential for this system to be used for scaled up production of Ag_2_S-NP and other inorganic nanoparticles. Furthermore, since the system described here was developed in accordance with industry standards for pharmaceutical synthesis, the transition to mass production will be easier. This study removes barriers to large animal studies and the eventual clinical use of Ag_2_S-NP in an innovative and potentially widely applicable manner.

One limitation to this study is the microfluidic chips used were not run to exhaustion, and therefore claims cannot be made about the longevity or reusability of the chips past the syntheses specifically described. However, when similar chips were used for lipid nanoparticle production, it was shown that they could be reused multiple times without compromising the product [29]. Additionally, though this work tests up to 256 parallelized channels and reagent concentrations increased up to 3000% of the original, higher throughput methods could still be explored, such as higher flow rates, although this can impact the characteristics of the resulting nanoparticles [54]. Furthermore, although it is expected that similar results would be observed with other inorganic nanoparticles, this has yet to be explored with the SSMS system. Finally, the contrast generation and biodistribution profile presented are limited to the µCT scanner and mouse model used.

In the future, it may be advantageous to develop a system for synthesis that includes formulation, filtration, and concentration of nanoparticles in one continuous system. To this end, it would be beneficial to evaluate the feasibility of incorporating a tangential flow filtration system in place of the current system of centrifugation with centrifugal filters. Further studies of the Ag_2_S-NP biodistribution, clearance, and safety could also be performed in large animal models, as the high-capacity synthesis methods presented herein have made these studies more feasible. It would also be valuable to evaluate the performance of these nanoparticles in a breast cancer disease model compared to that of an iodinated contrast agent, confirming that this contrast agent outperforms clinically used agents in vivo, as they have been shown to in vitro.

## Conclusion

The SSMS system can provide large-scale increase in production of Ag_2_S-NP without sacrificing nanoparticle quality or imaging utility. We demonstrated total production rates up to 17 L/hr when using a 256X channel chip, which is more than 100-fold higher than a single channel chip. This production increase could be further increased by use of multiple chips or 1000X channel chips, for example. Additionally, Ag_2_S-NP produced using this novel system retain their contrast and clearance properties in vivo. The best-in-class clearance rates of the Ag_2_S-NP, paired with significantly improved contrast generation compared to clinically available iodinated agents, make this contrast agent promising for further development and confirms the need for clinical scale production capacity. This study validates the potential for a substantial scale up in production, resulting in an improved outlook for clinical translation for Ag_2_S-NP.

## Supplementary Information

Below is the link to the electronic supplementary material.

Supplementary file1 Nanoparticle size and concentration over time.  Ag_2_S-NP zeta potential. X-Ray diffraction patterns. 2D µCT renderings of kidneys and bladder. Quantification of CT attenuation in heart and liver. Silver content retained in carcass and tail. (DOCX 1995 KB)
