# Supplementary material for: Towards the clinical translation of a silver sulfide nanoparticle contrast agent: large scale production with a highly parallelized microfluidic chip
Source: Eur J Nucl Med Mol Imaging. 2024 Nov 12;52(3):1177–88. doi: 10.1007/s00259-024-06967-5 (PMC11753937; doi:10.1007/s00259-024-06967-5)
Supplement: Supplementary file 1 — Supplementary file1 Nanoparticle size and concentration over time. Ag2S-NP zeta potential. X-Ray diffraction patterns. 2D µCT renderings of kidneys and bladder. Quantification of CT attenuation in heart and liver. Silver content retained in carcass and tail. (DOCX 1995 KB) [file 259_2024_6967_MOESM1_ESM.docx]

Supporting Information:

**Towards the Clinical Translation of a Silver Sulfide Nanoparticle Contrast Agent: Large Scale Production with a Highly Parallelized Microfluidic Chip**

Katherine J. Mossburg^1,2^*, Sarah J. Shepherd^1^*, Diego Barragan^2,3^, Nathaniel H. O^2,4,5^, Emily K. Berkow^2,6^, Portia S. N. Maidment^2^, Derick N. Rosario Berrios^2,7^, Jessica C. Hsu^1,2┼^, Michael J. Siedlik^8^, Sagar Yadavali^8^, Michael J. Mitchell^1,9^, David Issadore^1,10,11^**, David P. Cormode^1,2,9^**

^1^Department of Bioengineering, University of Pennsylvania, Philadelphia, PA, USA

^2^Department of Radiology, University of Pennsylvania, Philadelphia, PA, USA

^3^Department of Biology, University of Pennsylvania, Philadelphia, PA, USA

^4^Department of Pharmaceutical Sciences, St. Joseph’s University, Philadelphia, PA, USA

^5^Department of Physics, St. Joseph’s University, Philadelphia, PA, USA

^6^Department of Chemistry, University of Pennsylvania, Philadelphia, PA, USA

^7^Biochemistry and Molecular Biophysics Graduate Group, University of Pennsylvania, Philadelphia, PA, USA

^8^InfiniFluidics, Inc, Philadelphia, PA, USA

^9^ Cardiovascular Institute, Perelman School of Medicine, University of Pennsylvania, PA, USA

^10^ Department of Electrical and Systems Engineering, University of Pennsylvania, PA, USA

^11^ Department of Chemical and Biomolecular Engineering, University of Pennsylvania, PA, USA

^┼^This author’s current affiliation is the Departments of Radiology and Medical Physics, University of Wisconsin-Madison, Madison, WI, USA.

*These authors contributed equally to this work.

**Corresponding authors.

Emails: [issadore@seas.upenn.edu](mailto:issadore@seas.upenn.edu); [david.cormode@pennmedicine.upenn.edu](mailto:david.cormode@pennmedicine.upenn.edu)


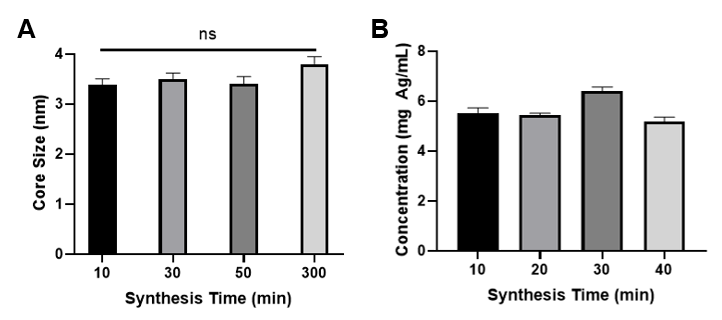


Figure S1. Ag_2_S-NP A) product size as evaluated by TEM and B) concentration as evaluated by ICP measured over time during synthesis by a 1X SSMS chip.


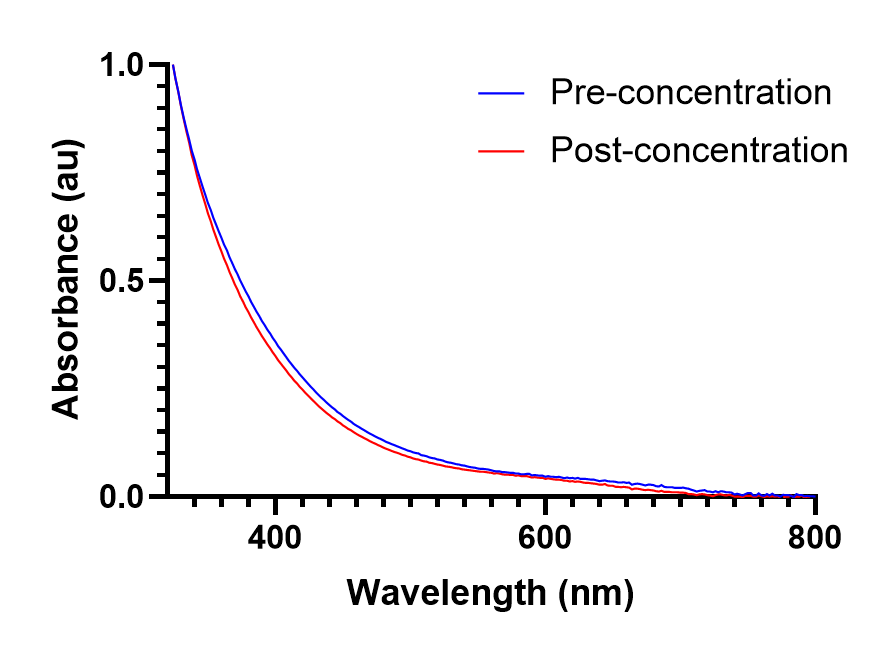


Figure S2. UV-visible absorption spectra of Ag2S-NP immediately after synthesis (pre-concentration) and after concentration and washing using molecular weight cut off tubes (post-concentration).


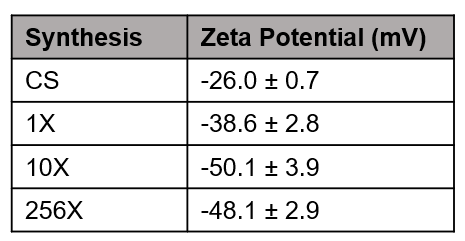


Figure S3. Table of zeta potential values for each Ag_2_S-NP synthetic condition.


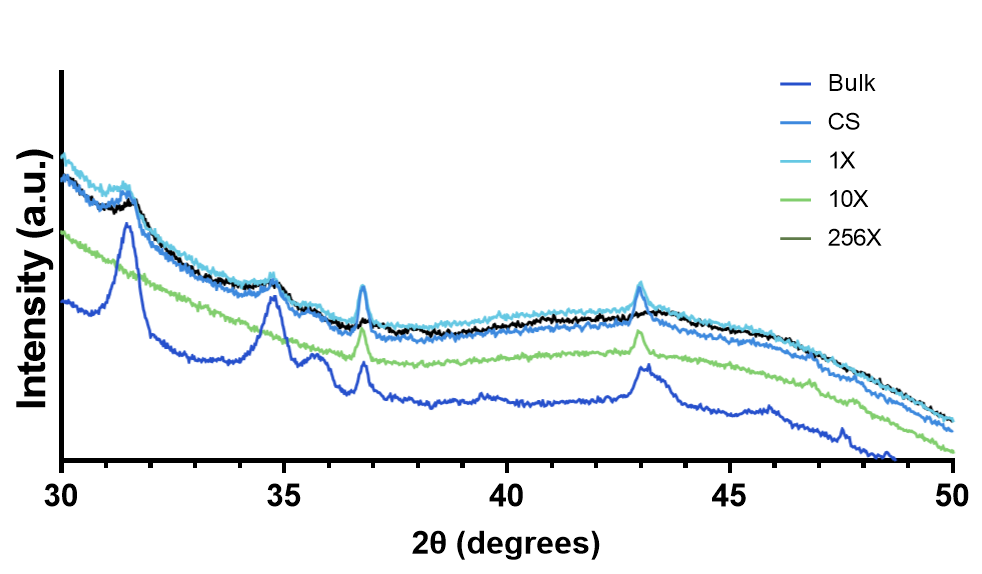


Figure S4. X-ray diffraction patterns of Ag_2_S-NP from each synthesis method.


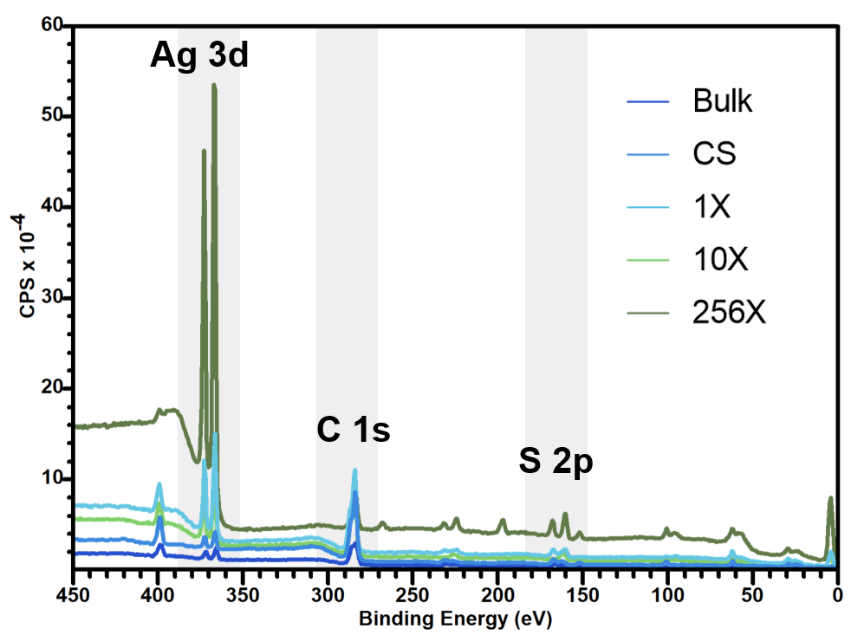


Figure S5. Survey XPS spectra of each type of Ag_2_S-NP.


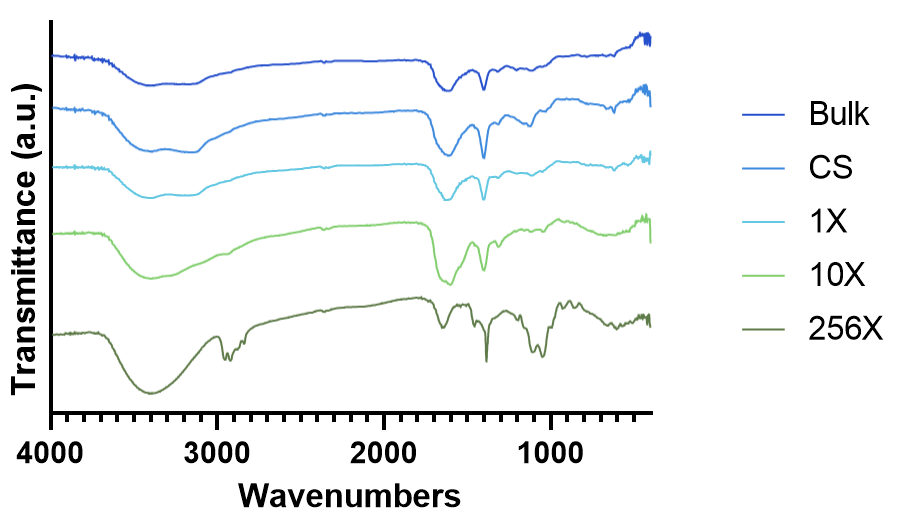


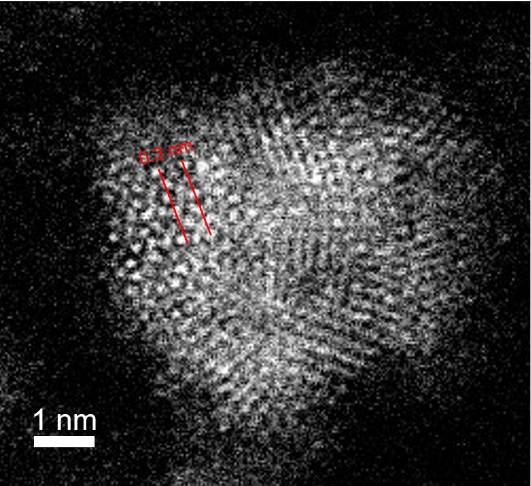
Figure S6. FT-IR spectra of each synthesis method of Ag_2_S-NP.

Figure S7. HR-TEM micrograph of representative CS-Ag_2_S-NP, showing 0.3 nm lattice fringe distance.


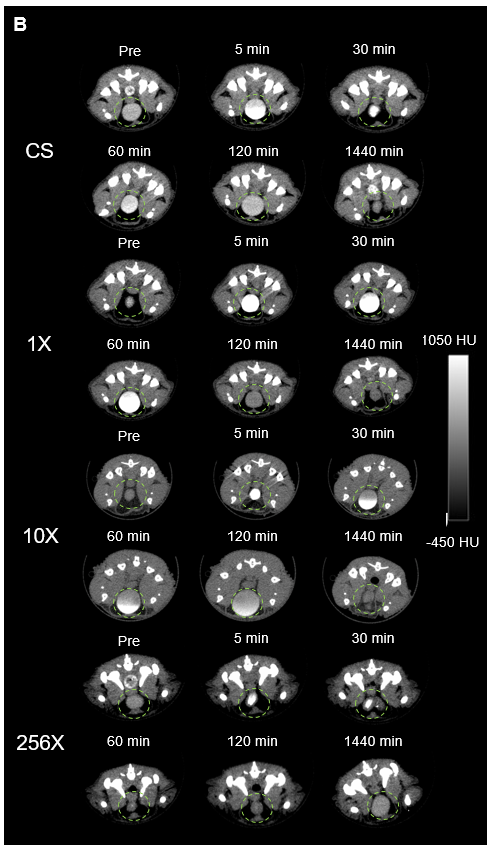

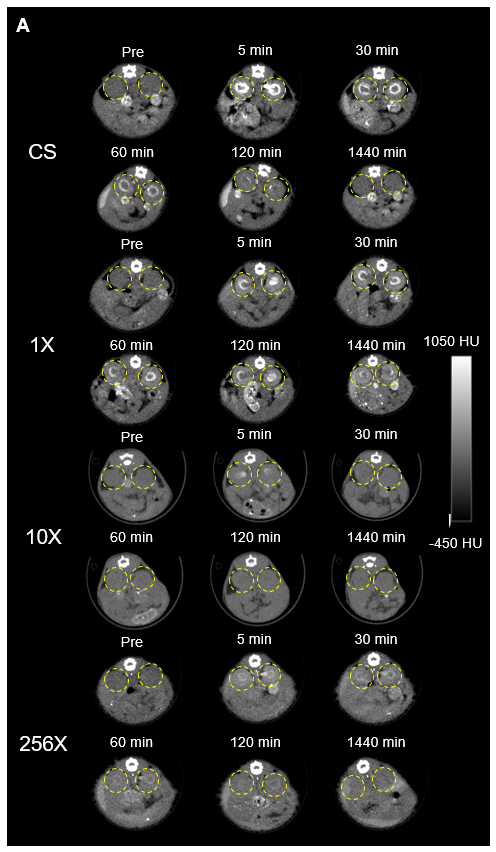


Figure S8. Representative µCT images showing Ag_2_S-NP being renally cleared through A) the kidneys (yellow circle) and B) the bladder (green circle).


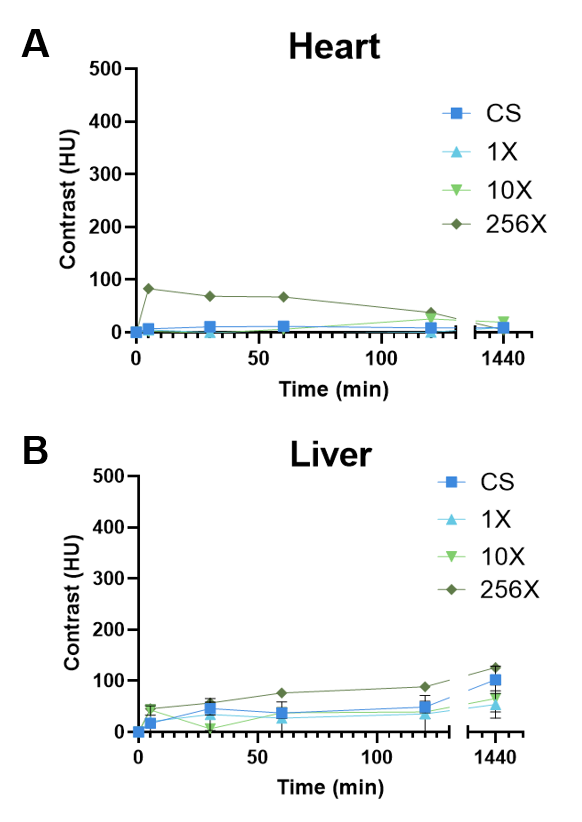


Figure S9. Quantification of CT attenuation in the A) heart and B) liver at each time point. n=5 per group. Data is presented as mean ± SEM.


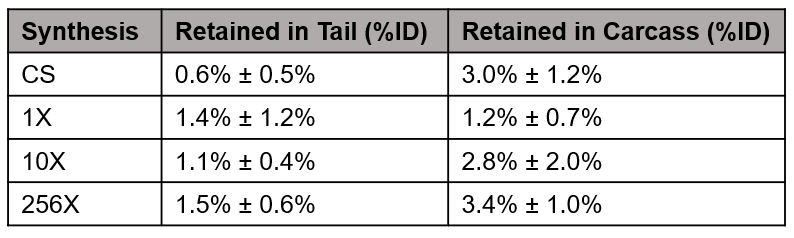


Figure S10. Silver content retained by the carcass and the tail of mice injected with Ag_2_SNP.
